# Application of Nanomaterials as an Advanced Strategy for the Diagnosis, Prevention, and Treatment of Viral Diseases

**DOI:** 10.3390/pharmaceutics13101570

**Published:** 2021-09-27

**Authors:** Jong-Woo Lim, Yu-Rim Ahn, Geunseon Park, Hyun-Ouk Kim, Seungjoo Haam

**Affiliations:** 1 Department of Chemical and Biomolecular Engineering, Yonsei University, 50 Yonsei-ro, Seoul 03722, Korea; lim333kr@hanmail.net (J.-W.L.); geunseon379@gmail.com (G.P.); 2 Division of Chemical Engineering and Bioengineering, College of Art, Culture and Engineering, Kangwon National University, Chuncheon-si 24341, Gangwon-do, Korea; reviana@kangwon.ac.kr; 3 Biohealth-machinery Convergence Engineering, Kangwon National University, Chuncheon-si 24341, Gangwon-do, Korea

**Keywords:** nanomaterials, viral diseases, vaccines, diagnosis, therapeutics

## Abstract

The coronavirus disease (COVID-19) pandemic poses serious global health concerns with the continued emergence of new variants. The periodic outbreak of novel emerging and re-emerging infectious pathogens has elevated concerns and challenges for the future. To develop mitigation strategies against infectious diseases, nano-based approaches are being increasingly applied in diagnostic systems, prophylactic vaccines, and therapeutics. This review presents the properties of various nanoplatforms and discusses their role in the development of sensors, vectors, delivery agents, intrinsic immunostimulants, and viral inhibitors. Advanced nanomedical applications for infectious diseases have been highlighted. Moreover, physicochemical properties that confer physiological advantages and contribute to the control and inhibition of infectious diseases have been discussed. Safety concerns limit the commercial production and clinical use of these technologies in humans; however, overcoming these limitations may enable the use of nanomaterials to resolve current infection control issues via application of nanomaterials as a platform for the diagnosis, prevention, and treatment of viral diseases.

## 1. Introduction

Since 2009, numerous infectious diseases have been occurring periodically, including those caused due to infection by influenza A virus (IAV; H1N1), West African Ebola virus, Middle East respiratory syndrome coronavirus (MERS-CoV), and the recent severe acute respiratory syndrome coronavirus 2 [SARS-CoV-2; known as coronavirus disease (COVID-19)]. SARS-CoV-2 has infected numerous individuals, which has resulted in millions of deaths. Owing to the rapid global spread of SARS-CoV-2 infections and the consequent damage, the World Health Organization (WHO) declared a COVID pandemic [1].

Strict measures, such as quarantining infected individuals, and technology-based approaches have been employed to prevent the further spread of COVID. Various types of healthcare technologies, including on-site rapid testing kits, vaccines, and antiviral therapeutics for COVID, have been developed and applied in a short period of time via Emergency Use Authorization (EUA) approvals. Most governments have carried out extensive testing to identify potential infections and have rolled out mass vaccination programs to prevent infection and reduce economic loss and human damage [2]. FDA-approved antiviral agents, such as remdesivir, have been used to treat SAR-CoV-2-infected patients, to reduce the number of severe cases that pose a serious burden on public health systems. It is now being recognized that the peak of the pandemic has passed, although the morbidity and mortality rates require further reduction. However, infection rates are fluctuating due to sporadic, rapidly rising infections with viral variants. The current pandemic situation is a potent warning indicating that the emergence or re-emergence of viral diseases is unpredictable but inevitable. Therefore, it is imperative to develop technology-based countermeasures for diagnosis, prevention, and treatment to cope with potential viral diseases in the future.

The introduction of nanomaterials in the development of technological countermeasures has advantages in many respects, such as facile functionalization, control of surface chemistry, and availability as a delivery carrier [3]. Nanomaterials can be tailored for specific uses by modulating physical and chemical properties, including size, morphology, surface charge, and solubility. Due to these controllable properties, nanomaterials have been used in biosensors to potentiate target-specific reactions that respond to biochemical environments, such as temperature, pH, and the presence of enzymes (Table 1) [4]. Furthermore, drug delivery carriers constructed with biocompatible nanomaterials have been actively researched to improve the efficacy of therapeutics, especially in cancer therapy. Nanomaterials can be applied in diagnosis, prophylaxis, and treatment systems to combat infectious diseases [5].

**Figure 1:**
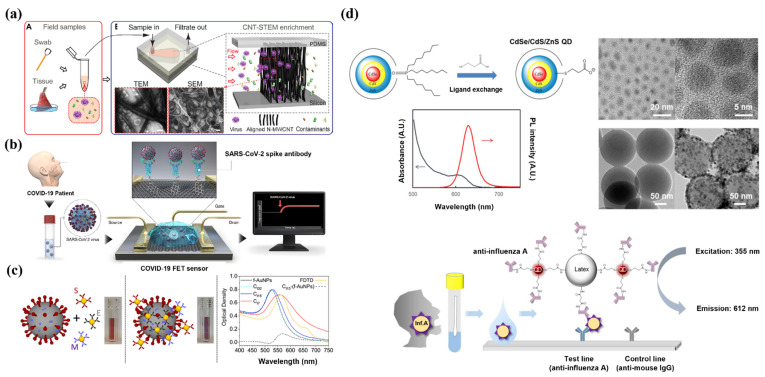
Application of nanomaterials for diagnosis with advanced sensitivity and selectivity. (**a**) Schematic of using carbon nanotubes for H5N2 isolation and concentration, directly from in situ samples. Transmission electron microscopy (TEM) and scanning electron microscopy (SEM) images of H5N2 separated by carbon nanotubes are shown. Reproduced with permission from [30]. Copyright (2016) American Association for Advancement of Science. (**b**) Schematic representation of graphene as a sensing material for detecting SARS-CoV-2. The SARS-CoV-2 spike antibody binds to graphene and the reaction with the target is converted into an electrochemical signal. Reproduced with permission from [31]. Copyright (2020) American Chemical Society. (**c**) AuNP-based colorimetric diagnosis of coronavirus disease (COVID-19). The surface-modified AuNPs with antibodies bind to the virus, which shifts the absorption wavelength of AuNPs. The shift in absorption wavelength changes the color of AuNPs from red to purple. Reproduced with permission from [32]. Copyright (2020) American Chemical Society. (**d**) Synthesis schematic of CdSe/CdS/ZnS quantum dots (QDs) and mechanism of application in rapid diagnostic strips. TEM images and fluorescence spectrum of synthesized QDs. Influenza A virus was detected using the fluorescence emission spectrum of the QDs on the rapid diagnostic strip. Reproduced with permission from [33]. Copyright (2020) Elsevier.

Herein, we review the potential of nanomaterials with respect to the development of technology for controlling viral diseases. First, we discuss rapid and sensitive detection systems based on nanomaterials using optical, electrochemical, and colorimetric signals (Figure 1). Next, we describe a nano-engineered vaccine system constructed with biocompatible materials and immune-stimulating compounds, eliciting potentially favorable immunogenicity [29]. Finally, we review antiviral nanosystems that potentiate the efficient delivery of antiviral therapeutics and exhibit virucidal activity. Thus, this review focuses on comprehensively exploring advanced nanomaterials for the diagnosis, prevention, and treatment of viral diseases, as well as their contribution to preparedness towards unpredictable viral diseases that may occur in the future.

## 2. Diagnosis

Diagnostic tests are a key component of any successful strategy aimed at suppressing emerging and re-emerging viral diseases and play an important role at all stages from early detection to final resolution [34]. Diagnostic tests appropriate for epidemic prevention and suppression are technically difficult to develop, validate, and implement and in-volve complex and time-consuming processes.

The accuracy of nucleic acid amplification tests is highly dependent on the time of obtaining the sample, as well as the type, storage, and handling of the sample. These tests can only diagnose active infections. False-negative results can occur if the sample is not obtained appropriately or if the subject is tested too soon or too late after exposure to the virus. In addition, the tests are complex and require specialized laboratory equipment and reagents, as well as specialists to perform the tests, which can be problematic due to the prolonged time required for obtaining the results.

Many researchers have attempted to overcome the limitations of reverse transcription polymerase chain reaction (RT-PCR) assays [35,36,37,38]. Antibody detection assays present several advantages over RT-PCR. Antibodies are more stable than RNA and are less degradable during transport and storage, thus reducing the risk of false-negative results. Although the research on antibody-based tests is ongoing, there are limitations to overcome. The main reason for this is the lack of specificity. Additionally, there is a lag phase from the initial virus exposure to the antibody response against infection. According to the accumulated immunological data, the antibody response peaks at approximately 11 days, indicating an insufficient time period for preventing the rapid spread of infectious diseases at early stages of infection. Therefore, antibody detection assays are less effective in diagnosing emerging and re-emerging viral diseases.

There is a need to develop new diagnostic platforms that are accurate, specific, fast, and easy to use, to facilitate rapid screening. Currently, research dynamics have shifted towards rapid diagnostics based on nanomaterials [39,40,41,42]. In this regard, nanotechnology-based applications can greatly improve the sensitivity of previously developed detection techniques, such as RT-PCR and immunoassays. Nanoparticles (NPs) have the characteristics of high adsorption capacity, the quantum size effect, and high reactivity. The large surface area of NPs can enhance detection effectiveness, as it allows efficient interaction with target analytes. Therefore, through physical or chemical bonding, nanomaterial-based diagnosis can be developed to increase selectivity and specificity and reduce detection time. Appropriately using advanced nanomaterials is the key to achieving improvements in nanotechnology. Nanomaterials are the basis for the design of a wide range of virus diagnostic tools. The unique characteristics of nanomaterials make them suitable for application in state-of-the-art virus detection technologies.

### 2.1. Carbon Nanotubes

Carbon nanotubes (CNTs) are hollow cylindrical structures with nano-sized diameters and are composed of sp2 sheets [43,44]. They are carbon-based nanomaterials that have excellent electrical conductivity and reactivity. Therefore, they can sense analytes and generate electrical signals as components of biosensors or improve recognition functions [45,46]. In addition, CNTs have large surface areas and high tensile strength because of the presence of sp2 bonds [47]. These nanomaterials are good candidates for nanoprobes and biosensors to detect viruses, owing to their unique electrochemical properties and chemical stability [48,49].

In a study on the detection of influenza A virus (IAV) using CNTs, a CNT-based DNA sensor was formulated by immobilizing probe DNA on 700 μm wide CNTs by simple physical adsorption. Owing to the excellent electrical conductivity and reactivity of CNTs, the reaction time for detecting the target DNA was less than one minute, and target samples of at least 1 pM were detectable [50]. In addition, after the first detection, the target DNA was removed, and reproducible results were reported in successive repeated measurements. Therefore, this CNT-based DNA sensor is expected to perform as an efficient virus diagnosis system because it can successfully detect IAV with high speed and a very low limit of detection (LOD), and can be reused [51].

In addition to biosensors, studies have reported that CNTs can be used to detect and concentrate viruses without labels due to their porous structure and high physical strength (Figure 1A) [52]. We developed CNT size-tunable enrichment microdevice (CNT-STEM) technology by bonding a multi-walled CNT with very high porosity to a poly (dimethylsiloxane) chamber. It has been reported that vertically aligned multiwall CNTs detect viruses in a viable state and can separate them from contaminants present in the sample. This system has enabled the detection of the H5N2 virus at a detection limit that is more than 100 times lower than that of real-time RT-PCR (rRT-PCR). The pore size of CNTs can be adjusted, resulting in selective virus detection and concentration. This system has the advantage of being able to capture viruses without labeling, enabling rapid detection of viruses in the field without the need for an amplification process [30].

### 2.2. Graphene

Graphene is composed of a single two-dimensional layer in which carbon atoms are covalently connected in a honeycomb lattice structure [30,53]. Graphene is widely used biologically because it allows easy immobilization of various biomolecules, such as nucleic acids and proteins, and its large surface area confers an advantage for the adsorption of analytes [53]. Conjugation with other materials increases the sensitivity of graphene, and this technique has been applied in pathogen detection systems [54,55]. Moreover, graphene is a conductive material with high electron mobility and chemical stability [56,57]. Therefore, it is possible to detect the interaction of the target material with a receptor immobilized on graphene. These properties make graphene particularly useful in biosensors that detect various viruses [58].

Recently, a graphene-based immunological diagnostic method was reported for SARS-CoV-2 (Figure 1B), which was used to detect the antigen protein via a conjugated SARS-CoV-2 spike antibody. The application of graphene resulted in an optimal sensing environment with improved sensitivity. This system exhibited a lower LOD (1 fg/mL) compared to the enzyme-linked immunosorbent assay. In addition, pre-treatment was not needed, and the analysis time was less than three minutes, thereby enabling real-time diagnosis. When a MERS-CoV protein was applied in combination with the target SARS-CoV-2 protein, the electrical signal of the latter was dominant. Therefore, this graphene-based system is expected perform as a point-of-care diagnostic system that can efficiently detect SARS-CoV-2 in real time with excellent signal detection ability and high selectivity [31].

### 2.3. Gold NPs (AuNPs)

AuNPs have a unique absorption spectrum in the visible region due to their surface plasmon characteristics. These characteristics are associated with the size, shape, and morphology of the AuNP. The surface free electron arrangement of AuNPs changes in response to the physicochemical environment, such as the presence of redox agents, pH, and physical binding. AuNP responses involve color changes, thus making them potential nanomaterials for a diagnostic platform that is based on optical signals. Another characteristic of AuNPs is their amenability to easy surface modification. Au–thiol chemistry facilitates easy and robust covalent bonding of the AuNPs with various compounds of interest, such as probes, antigens, and antibodies [59,60,61]. Thus, surface-modified AuNPs, engineered to bind to target molecules, can facilitate target-specific detection with the naked eye [62].

Color changes occurring due to the binding of the AuNP to the target are rapid, and the optical response of the AuNP can be interpreted visually; this is an important characteristic that contributes to the rapidity of viral detection. For example, a colorimetric diagnostic detection system based on AuNPs labeled with three types of antibodies against a surface protein of SARS-CoV-2 has been reported (Figure 1C). When the antibody-labeled AuNP is mixed with the SARS-CoV-2 surface protein, the binding of the AuNP to the target protein results in a change in the absorption wavelength of the AuNP. This results in a color shift and is achieved within three minutes. This system demonstrates the effectiveness of AuNPs as part of a rapid diagnostic platform enabling the detection of SARS-CoV-2 without sophisticated instrumentation [32].

A lateral flow system associated with AuNPs is currently being used as a rapid diagnostic device. This device captures target molecules in the flowing specimen by capillary action, and the results are immediately visualized on a strip labeled with antibodies [63]. This rapid diagnostic system combined with a simple detection procedure and easy read-out with the naked eye enables portable detection of the virus outside the laboratory [64]. This simple method of detecting a target depending on the presence or absence of a red line, and the rapid and visual diagnosis of the results, are valuable in field diagnoses, especially when the sample size is large. A recent study has reported a lateral flow-based rapid diagnostic system for SARS-CoV-2 that uses antigen-antibody reaction. This system uses surface-modified AuNPs linked to anti-human IgM antibodies that capture SARS-CoV-2 viral particles via antigen-antibody reaction, and the results are evaluated based on the appearance of a red line [65]. In addition, the application of AuNP-based lateral flow systems is not limited to antigen–antibody reaction-based detection, and surface modification of AuNPs has been widely used to detect genetic material. For example, a AuNP-based dengue virus (DENV) detection system has recently been reported. AuNPs combined with the reporter probe (rDNA) are prepared using a simple modification process to detect target genetic material via a nucleic acid sandwich reaction. AuNP-rDNA interacts easily with the target DENV-1 RNA in lateral flow strips; the occurrence of this interaction is confirmed via the appearance of a red line. This system demonstrated an analysis time of less than 20 min for the detection of DENV [66]. AuNP-based lateral flow can be applied to point-of-care testing because it can be utilized outside the laboratory; the associated procedure is simple and consumes a small amount of sample, and there is a minimal requirement for equipment [67]. In addition, AuNPs can be applied to PCR (which is the gold standard for rapid diagnosis) to reduce analysis time and improve sensitivity. Another study reported the use of spherical nucleic acids (SNAs) with AuNPs to improve the detection of SARS-CoV-2 by conventional PCR. SNAs attached to the surface of AuNPs form novel three-dimensional nanostructures with nucleic acids that are complementary to linker-DNA probes (binding occurs via covalent bonding) [68]. Target-dependent cleavage of the linker-DNA probes results in the aggregation of AuNP-SNAs, leading to a colorimetric change. The color change occurs within 10 minutes of mixing the PCR product with AuNP-SNAs, and the sensitivity is comparable to that of RT-PCR [69]. A simple PCR- and AuNP-SNA-based colorimetric analysis can contribute as a complementary technique to RT-PCR, to shorten detection time by simplifying the analysis process. Therefore, this system can serve as a powerful diagnostic tool for infectious diseases and will help reduce the burden on real-time RT-PCR testing. In conclusion, AuNPs can play a pivotal role in the development of detection systems for various types of viruses. Certain properties, such as ease of surface functionalization and visualization, aid in the development of rapid diagnostics that ultimately contribute to minimizing the spread of viral diseases at the early stages [70].

### 2.4. Quantum Dots (QDs)

QDs are being applied in various fields, such as diagnosis and biosensing, owing to their unique optical and electrical properties. QDs characteristically show various emission wavelengths and colors, depending on their size and shape [71,72,73,74]. However, as electrons are easily lost from QDs because of their high energy states, a structure in which a polymer coats the core and shell is mainly used [75,76]. This core/shell structure has a long persistence and high photoluminescence efficiency. QDs are inorganic fluorophores that show minimal reactivity with surrounding materials, which results in high photostability [33,77]. Due to these optical properties, QDs are strongly fluorescent even when illuminated with a single light source. Therefore, they can be applied as visualization tools in systems that detect and label viruses [74].

A CdSe/CdS/ZnS QD-based rapid fluorescence immunochromatographic test (FICT) has been reported for detecting the IAV (Figure 1D). By combining QDs with anti-influenza A antibodies, a QD complex was formulated to detect H1N1 and H3N2 in clinical samples. The QD complex reported a sensitivity of 93.75%. This sensitivity was higher than that of the europium NP-based FICT, which reported a clinical sensitivity of about 79%. The QD complex also performed eight times better than europium NPs and 64 times better than a rapid diagnostic test. The aforementioned study reported the rapid detection of two subtypes of IAV (H1N1 and H3N2) with superior sensitivity using QDs [78].

QDs can also be used to detect hepatitis B virus (HBV) gene mutations due to their excellent sensitivity. When a streptavidin-labeled QD binds to HBV DNA, it emits a green fluorescence signal with a wavelength of 460–550 nm, which can be detected via microscopy. This system exhibited a high sensitivity and very low LOD. HBV mutant detection is simple and rapid with this system, showing excellent signal strength and photostability without the need for additional procedures [79].

### 2.5. Synthetic Polymers

Synthetic polymers have been widely used in diagnostic fields, and those with biocompatibility and biodegradability have contributed to advancements in the biomedical field. The convergence of biotechnology and diagnostic technology has brought forth a need for biomedical materials and novel synthetic polymers with unique properties [80,81,82]. Polylactic acid (PLA) is a synthetic polymer characterized by biodegradability, biocompatibility, and ease of manufacture [83]. Due to its polymeric properties, it can be used to prepare biocompatible membranes. These membranes show improved physical properties, such as increased tensile strength. Additionally, the rate of decomposition and ion release can be controlled according to the surrounding environment [84]. PLA can also help improve adhesion and the stability of metallic materials [85]. In this regard, several types of synthetic polymers use an electrospinning process. For example, polyvinyl alcohol (PVA) is a biocompatible, soluble, linear synthetic polymer that has a variety of uses in biomedical applications [86]. Its tension-activated properties facilitate the electrospinning process, enabling the application of metallic nanomaterials. PVA has excellent film-forming ability and physical and chemical strength. Due to these properties, PVA is often used in the development of diagnostic systems. Other conjugated polymers with unique colorimetric and optical properties, such as polydiacetylene (PDA) and polyaniline, are also used as nanomaterials [87,88].

PDA reacts to environmental changes, such as a change in pH, and chemical and biological stimuli. Due to these unique properties, PDA changes color in response to the binding of the target to the receptor, allowing it to be used to detect viruses [89]. For example, a PDA-based biosensor for the detection of influenza virus has been reported. A PDA vesicle formed with an optimized ratio of 10,12-pentacosadiynoic acid and 1,2-dimyristoyl-sn-glycero-3-phosphocholine was designed for the rapid detection of H5 influenza virus. A change in color, from blue to red, was visible upon detection of H5 influenza virus by the PDA-based biosensor within 20 minutes. Specificity towards various viruses, such as H3 influenza virus, Newcastle disease virus strain, and a porcine reproductive and respiratory syndrome virus strain, was tested (at the same concentration). However, the PDA-based biosensor exhibited a color change only in response to the H5 influenza virus. These results demonstrated that the PDA biosensor could specifically discriminate H5 influenza virus from non-target viruses. In addition, the sensitivity was 13.5 copies/µL, which is considered an outstanding performance. This diagnostic system is simple and rapid, and it allows for easy visualization and interpretation of the results with the naked eye. This system may overcome certain limitations in existing detection methods, demonstrating the potential of PDA-based diagnostic systems in point-of-care testing [90].

Polyaniline (PANI) can act as an electron transfer mediator, owing to its unique chemical structure, and thus, can exhibit high conductivity and redox activity, as per the pH of the surroundings. PANI is useful for biosensor development because it has excellent electrochemical and mechanical stability, which make it suitable for structurally binding various biomolecules [91]. Recently, a PANI-based immunological diagnostic system for DENV was reported. This electrochemical immunosensor for DENV was constructed by immobilizing an antibody on PANI bound to the surface of a glassy carbon electrode. The conductive polymer, PANI, converted DENV NS1 antigen–antibody reactions into an electrical signal. This immunosensor achieved a wide detection range, from 1 ng/mL to 100 ng/mL, with an LOD of 0.33 ng/mL. These results show that the PANI-based immunosensor demonstrates the potential to detect DENV with excellent sensitivity and via successful signal transduction of antigen–antibody reactions. This technology does not require additional labeling, which can reduce time and manufacturing costs. Therefore, conductive polymer-based immunosensors may be used to increase the efficiency of on-site diagnosis of DENV [92].

## 3. NP Vaccines for Emerging Viruses

**Figure 2:**
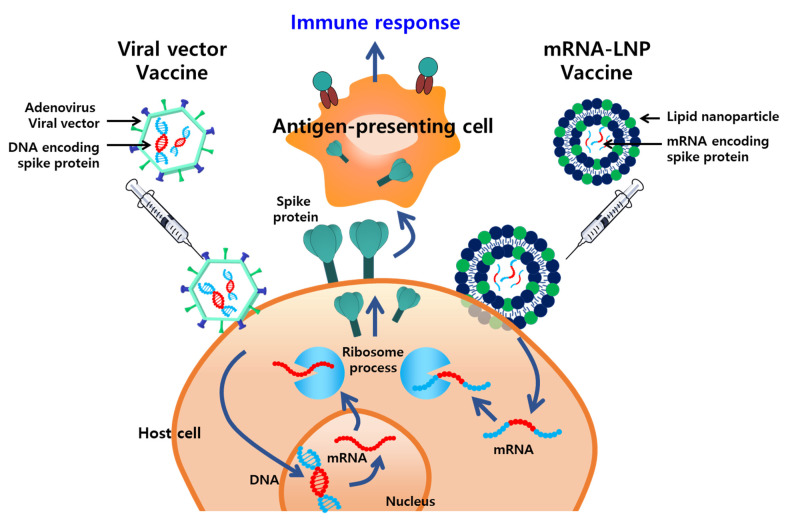
Overview of nanoplatform-based vaccines approved by the World Health Organization (WHO) for the prevention of emerging infectious diseases. Viral vectors and lipid NPs (LNPs) elicit potent immune responses by stably delivering DNA and mRNA encoding antigens, respectively, to antigen-presenting cells.

Vaccines are the most effective and cost-efficient means for preventing infectious diseases. Despite the significant successes of various vaccines, the ongoing development of new, safer, and more potent vaccines is required because of the emergence of new pathogens, recurrence of old pathogens, and mutations in existing pathogens. Typically, vaccines incorporate adjuvants, which are supplementary substances that compensate for the poor immunogenicity of antigens and enhance the cellular and humoral immunity. Several nanoplatforms that improve vaccine immunogenicity by enhancing the delivery of antigens to the immune system or via a depot effect have been developed. The WHO Emergency Use Listing for combating the COVID-19 pandemic includes vaccines that incorporate a delivery system based on NPs, such as lipid NPs (LNPs) (Moderna, MA, US and Pfizer-BioNTech, NY, US) and adenovirus viral vectors (Janssen, NJ, US and AstraZeneca, UK) (Figure 2).

Delivery systems formulated with NPs are being used in next-generation vaccines for effectively delivering antigens and/or intrinsic immunostimulants [93,94,95,96,97]. These vaccines are being engineered not only for the prevention of emerging infectious diseases, but also for treating chronic diseases, such as diseases caused by hepatitis C infections [98,99], human immunodeficiency virus (HIV) infections [100,101], herpes [102], and cancer. Next-generation vaccines aim to induce both humoral and cellular immune responses, while being applied prophylactically and therapeutically [103,104]. Various nanoplatforms have been incorporated into delivery systems and immunogenicity-enhancing strategies, such as LNPs, polymeric NPs, nano-complexes, virus-like particles, and inorganic NPs. The main principle of delivery systems is to deliver the vaccine antigens or immunopotentiators to the antigen-presenting cells (APCs; including macrophages and dendritic cells) responsible for the induction of innate immune responses. A delivery system that is similar in size to nano-sized pathogens is expected to be advantageous for APC phagocytosis and for presenting antigens to naïve T cells in lymphoid tissues. Further, nanoparticulate delivery agents can protect the payload, deliver it in an intact native conformation to a target site within the immune system, and create a sustained release of antigens over time. In the future, the usage of such next-generation nano-delivery systems in vaccine technology is expected continue with sufficient safety and stability. Herein, we discuss the use of several representative nanoplatforms (LNPs and polymer particles) in delivery systems that enhance vaccine efficacy. We also review nanotechnology-based adjuvants that enhance the intensity and quality of cellular and humoral immune responses for the development of vaccines.

### 3.1. LNPs

Nucleic acid-based vaccine technologies developed using plasmid DNA and mRNA have emerged as potential alternatives to conventional vaccines. mRNA vaccines function as non-infectious antigens, owing to which the risk of infection and subsequent transmission is eliminated. As mRNA molecules are fragile and are easily degraded during normal cellular processes, special delivery methods or LNPs are used to secure stability and to deliver the molecules to the cytoplasm for transcription. LNPs have been widely used as mRNA delivery agents for decades. Generally, they consist of four components, namely, lipids, phospholipids, poly (ethylene glycol) (PEG), and cholesterol, which support self-assembly into NPs (~100 nm) with bilayer membrane-like structures. These components affect the flexibility and stability of the structure and prolong the half-life of the structure by preventing the degradation of proteins and enzymes. The first mRNA vaccine entrapped in LNPs was developed in 1993 against the influenza virus. Thereafter, LNPs were mainly used for delivering clustered, regularly interspaced short palindromic repeats (CRISPR)-Cas9 RNA; neo-antigens; and tumor-associated antigen mRNAs for cancer therapeutic vaccines. In addition, recent studies have been conducted to utilize bioactive lipids such as plasmalogen for therapeutic, prophylactic components as well as delivery agents [105]. Given the recent clinical success of mRNA-LNP vaccines and the dramatic efficacy of these vaccines against COVID-19, many institutions worldwide are studying and developing these systems to create next-generation vaccines.

mRNA delivery for efficient translation requires cellular uptake and escape from the endosome into the cytosol of APCs. LNPs facilitate efficient uptake of the mRNA and prolonged interaction with the target cells; moreover, they protect the mRNA from being degraded in the cellular environment (5′ and 3′ exonucleases, endonucleases, and pH). The liposomal delivery systems usually use cationic lipids, such as DOTMA (1,2-di-O-octadecenyl-3-trimethylammonium-propane) and DOTAP (1,2-dioleoyl-3-trimethylammonium-propane), which readily form complexes with anionic mRNA. Repulsion between an mRNA molecule and an anionic cell membrane reduces the uptake rate of the mRNA. The electrostatic interactions between cationic LNPs and mRNA molecules ensure stable intracellular delivery [106,107]. Additionally, cationic lipid components induce prolonged retention at the injection sites and activation of innate immunity, including both Th1- and Th2-type immune responses [108,109,110].

Potent immunogenicity was observed after intradermal injection of mice with cationic LNPs (~80 nm) encapsulating mRNAs that encoded the Zika virus target antigen (ZIKV-LNP) [111]. ZIKV envelope protein-specific CD4+ T cell activation and the enhanced induction of cytokines interferon (IFN)-γ, tumor necrosis factor (TNF)-α, and interleukin (IL)-2 in re-stimulated splenocytes were observed two weeks after administering a single dose of the vaccine (30 μg of ZIKV prM-E mRNA-LNP) to mice. Potent antigen-specific antibody responses and ZIKV-neutralizing activity were observed in C57BL/6 mice at 8–12 weeks and in BALB/c mice at 2–20 weeks. Additionally, intradermal immunization with 50 μg of ZIKV prM-E mRNA-LNP induced a significant production of antigen-specific antibodies and resulted in neutralizing efficacy and protection against ZIKV challenges, five weeks after vaccinating a non-human primate species (rhesus macaques).

Furthermore, Perrie et al. investigated and compared various cationic lipids, namely, 3ß-[*N*-(*N*’,*N*’-dimethylaminoethane)-carbamoyl]cholesterol (DC-Chol), dimethyldioctadecylammonium (DDA), 1,2-dioleoyl-3-trimethylammonium-propane (DOTAP), 1,2-dimyristoyl-3-trimethylammonium-propane (DMTAP), 1,2-stearoyl-3-trimethylammonium-propane (DSTAP), and *N*-(4-carboxybenzyl)-*N*,*N*-dimethyl-2,3-bis (oleoyloxy)propan-1-aminium (DOBAQ), with respect to efficient mRNA transfection and antigen expression induction [112]. The cationic lipid-formulated NPs included the following combinations: DOPE and DMG-PEG2000 or DSPC; and Chol and DMG-PEG2000. Self-amplifying RNA (SAM) encoding the rabies virus glycoprotein (RVG) was used as the nucleic acid antigen. Cationic LNPs, including combinations with DOPE and DOTAP or DDA, induced the most potent in vitro RVG expression in the presence or absence of 5% FBS. Furthermore, DOTAP- or DDA-LNP formulations induced efficient production of RVG-specific antibodies and cytokine-producing CD8+ and CD4+ T cells in vivo. Both cationic formulations and lipofectamine 2000 (a proprietary cationic lipid formulation), which has been widely used in clinical studies, induced significant humoral and cellular immunity in mice when complexed with SAM at high doses. These results revealed a useful alternative to ionizable lipids for the delivery of SAM vaccines.

Although the properties of LNPs for vaccine delivery are suitable for clinical application, there are a few safety concerns that need to be addressed. Despite the associated desirable outcomes, such as efficient delivery and translation of anionic nucleic acids, cationic lipids have been associated with cellular toxicity and inflammatory responses. For example, cationic LNPs have induced several changes in cells, including cell shrinking, reduced mitosis, and vacuolization of the cytoplasm, followed by cell lysis and necrosis [113,114,115]. The capture of LNPs by Kupffer cells in the liver can trigger excessive inflammation by inducing the expression of cytokines (TNF-α, IFN-γ, IL-6, and IL-12), which may cause liver damage [116]. In addition, several components of LNPs (e.g., PEG, hydrophobic chains, hydrophilic groups, and linker bonds) can potentially promote local and systemic toxicities. Consequently, achieving a balance between acceptable toxicity and good performance remains a key challenge in the development of LNP-based vaccines [117].

### 3.2. Polymeric NPs

To enhance the immunogenicity of vaccines, several different types of nano-vehicles other than LNPs have been used as carriers for antigens or other agents, such as antibodies, ligands, and immune-stimulators. Specifically, biocompatible and biodegradable polymeric NPs, such as polymersomes, micelles, nanospheres, and hydrogels, are potent delivery vectors for modulating vaccine-induced immune responses. Polymeric NPs provide various options to enhance the activation of APCs, increase presentation of antigens on both MHC class I and II molecules, prolong antigen release, induce long-lasting immune responses, and increase neutralization efficacy. These properties are vital for ensuring protection against infections. The advantages of using polymeric NPs in vaccines are associated with their physicochemical properties (size, shape, rigidity, ionization, and protein conjugation), which can be custom engineered for application in extra- and intracellular environments.

**Figure 3:**
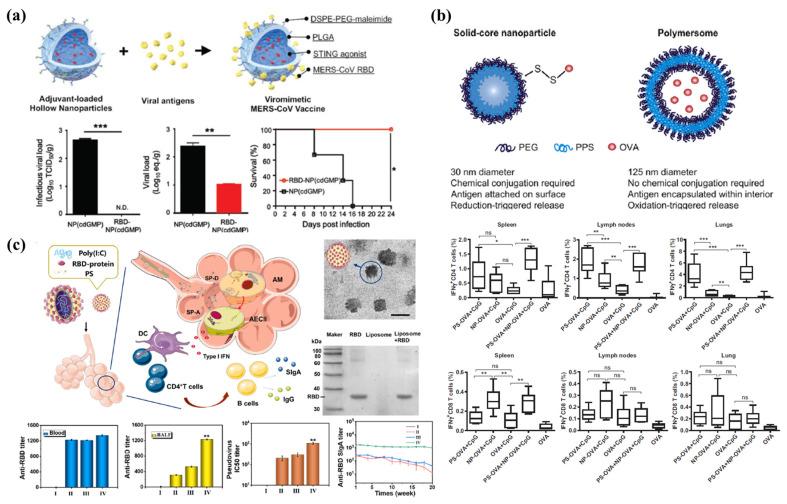
Antigen and immunostimulant delivery using nanoparticles. (**a**) Schematic showing the preparation of a viromimetic nanoparticle vaccine using hollow PLGA nanoparticles with encapsulated adjuvant and surface maleimide linkers for conjugating viral antigens. Protection efficiency of vaccinated hDPP4 transgenic mice against lethal infection with Middle East respiratory syndrome coronavirus (MERS-CoV). Statistical analyses were performed by unpaired *t*-tests (* *p* < 0.05, ** *p* < 0.01, *** *p* < 0.001). Reproduced with permission from [120]. Copyright (2019) Wiley. (**b**) Schematic of solid-core NP and watery-core polymersome (PS). Proportions of IFNγ-producing CD4+ and CD8+ T cells in the spleen, lymph nodes, and lungs after immunization with NP and PS. The non-parametric Manne-Whitney *U-test* was used to compare experimental groups (* *p* < 0.05; ** *p* < 0.01; *** *p* < 0.001; ns, not significant). Reproduced with permission from [121]. Copyright (2013) Elsevier. (**c**) Schematic showing inhalable bionic-virus nanovaccine activating cellular immunity and humoral immunity of respiratory mucosa. TEM images of bionic-virus particles. Scale bars, 200nm. Antigen-specific immune responses (IgG, IgA titer, and inhibition titer) upon immunization with inhalable bionic-virus nanovaccine. (I: PBS, II: Intramuscular Injection, III: Intraperitoneal Injection, IV: Nasal Delivery) Reproduced with permission from [122]. Copyright (2021) Elsevier.

In vaccine development, nanoparticulate vaccines have been customized to mimic viruses, with respect to morphology and multivalent epitope exhibition, as well as to co-deliver and release immune-stimulants [118,119,120]. PLGA NPs (~120 nm) have been used extensively to formulate viromimetic matrices with hollow aqueous cores to entrap the soluble stimulator of interferon gene (STING) agonist adjuvants and can be engineered to have a thin shell (~10 nm) to enable conjugation of viral antigens (Figure 3A) [120]. Hollow polymeric NPs have been prepared with PLGA and DSPE-PEG-maleimide via water-in-oil-in-water double emulsion methods. Upon subsequent antigen conjugation and STING agonist encapsulation, the quantities of the loaded agonist and MERS-CoV RBD proteins were analyzed using high performance liquid chromatography (HPLC) and bicinchoninic acid (BCA) assays, respectively. Antibody-conjugated colloidal gold was used for staining, to enable the visualization of the conjugated antigens. Adjuvant-loaded MERS-CoV viromimetic NP vaccines showed pH-responsive release under acidic conditions (pH 5) in endosomes and were effective for the co-delivery of antigens and STING agonists to drain lymph nodes. MERS-CoV vaccines elicited prominent immune responses in vivo, including potent neutralization efficacy and antigen-specific T cell responses. These vaccines further conferred protection against lethal challenges associated with MERS-CoV infection in transgenic mouse models.

The conjugation of antigens and TLR agonists to the delivery vehicles has been shown to elicit enhanced immune responses in some cases. However, the type of association method (conjugation or encapsulation) may affect the extent of immune responses [121]. The co-encapsulation of antigens and MPLA in polymersomes significantly increased antigen-specific antibody production and cytokine expression levels compared to those achieved via the association of antigens and MPLA without vehicles [123]. Associating antigens with nanoparticulate systems may also promote immune responses mediated by T helper or cytotoxic T lymphocytes. In the PEG-b-PPS polymer compositions, antigen-conjugated solid core NPs (30 nm) showed prevalent CD8+ T cell responses in the spleen, lymph nodes, and lungs, whereas antigen-loaded polymersomes induced enhanced antigen-specific CD4+ T cell responses (Figure 3B). The ability to selectively elicit different T cell responses using different nanoparticulate antigen delivery systems could be valuable for proactive vaccine design; it would also enable modulation of naïve immune systems to combat emerging and re-emerging infectious diseases.

Mucosal vaccines present a potential strategy for inducing potent immune responses, suppressing infection, and preventing viral transmission. As pathogens generally invade the host through the mucosa, inducing protective immunity in the mucosal routes of infection is the most effective preventive measure for reducing the incidence of mucosally transmitted infectious diseases. Specifically, the nasal administration strategy has been effective for inducing mucosal immunity against respiratory tract-based infectious diseases. The administration of mucosal vaccines leads to the production of antigen-specific immunoglobulin A (IgA), which neutralizes invading pathogens and protects against lethal infections; consequently, viral binding to target cells is inhibited and viral proliferation is suppressed. Ming et al. suggested the formulation of a biomimetic coronavirus to imitate the structure of the pathogen and utilization of the pathogen-specific route of infection. This biomimetic formulation has been proposed as a potential mucosal immunity-inducing vaccine [122]. Biomimetic pulmonary surfactants and PEG-lipid-based virosomes were conjugated with the receptor binding domains of the multivalent spike protein of SARS-CoV-2 to simulate the structure of coronaviruses (Figure 3C). Poly (I:C) is a synthetic double-stranded RNA that activates the toll-like receptor 3 pathway to induce strong innate immunity; this molecule was encapsulated in virosomes to formulate a nano-virosome vaccine. This vaccine can be administrated intranasally to imitate a COVID-19 infection. It demonstrated the capacity to induce a high titer of IgA and a potent neutralization efficacy against SARS-CoV-2. In addition, many published studies have shown that intranasally administrated ionic polymeric NPs induce an immune response by overcoming mucociliary clearance and delivering antigens within the epithelial barrier [124,125,126]. The mucosal route of administration is a non-invasive route of vaccine delivery and enables the induction of both systemic and mucosal immunity against mucosal infectious pathogens such as coronaviruses, influenza viruses, herpes simplex virus type 2, and HIV type 1 (HIV-1).

### 3.3. Nanoparticulate Adjuvants

Most adjuvants in advanced development focus on nanoparticulate platforms and immunomodulatory properties [127]. MF59 is a well-known adjuvanted seasonal flu vaccine (Fluad^TM^) and was approved in Europe in 1997. This vaccine is composed of nano-sized squalene droplets with biocompatible surfactants (Tween 80 and Span 85) in a citrate buffer. Although the specific mechanisms of action of emulsions are incompletely understood, emulsions are known to induce the recruitment of CD11b+ inflammatory cells to the injection site and the production of a broad range of cytokines and chemokines. Additionally, they control the release rate of the antigen (depot effect) for enhanced and consistent induction of immune responses [128]. Alternative squalene-based nano-emulsions, such as AF03 and AS03, have been developed with additional excipients [129,130]. These formulations have long-term stability and elicit robust innate immune and antibody responses.

**Figure 4:**
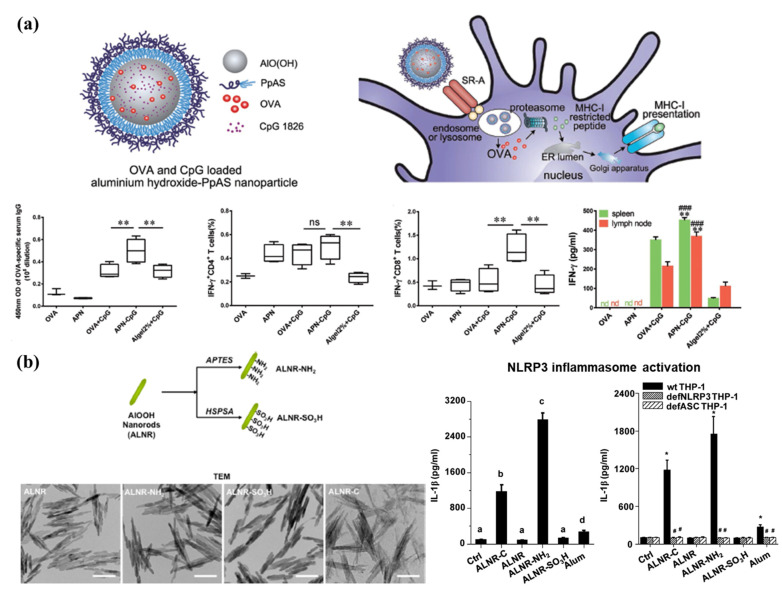
Nanoparticulate aluminum-based adjuvants. (**a**) Schematic representation of aluminum hydroxide-PpAS nanoparticles (APNs) adsorbing OVA and CpG. APNs promote antigen cross-presentation via a cytosolic pathway in dendritic cells. Enhanced antigen-specific humoral and cellular immune responses after immunization with APNs. Statistical analysis was performed using one-way ANOVA, followed by the Bonferroni post-test. * *p* < 0.05; ** *p* < 0.01; ^#^ *p* < 0.05; ^###^ *p* < 0.001; ns, not significant; nd, not detectable. Reproduced with permission from [132]. Copyright (2018) American Association for Advancement of Science. (**b**) Schematic of the surface charge functionalization of aluminum oxyhydroxide [AlO(OH)] with APTES and HSPSA. Representative TEM images of ALNR, ALNR-NH2, ALNR-SO3H and ALNR-C, where the scale bars was 200 nm. NLRP3 inflammasome activation after treatment with ALNR has also been shown. Statistical analysis was performed using Tukey’s test. The values that do not share the same letter indicate statistical differences at *p* < 0.01. Reproduced with permission from [131]. Copyright (2017) American Chemical Society.

Alum has been administered as a safe and effective vaccine adjuvant to diverse populations globally over the past 80 years. It has been licensed for use as an adjuvant in the clinic; however, its primary limitation is that it does not elicit Th1 responses. Alum formulated as micro-sized gels has been used widely as a vaccine carrier to adsorb antigens. Some studies have elucidated that the physicochemical characteristics (particle size, surface charge, and shape) of the formulation affect the biological activity of the vaccines [131,132,133]. The MHC-I pathway is essential to induce a strong CD8+ T cell-mediated immune response. However, exogenous antigens proceed through MHC-II presentation. This can be improved by involving the cytosolic endocytosis pathway within APCs. Jiang et al. demonstrated that aluminum oxyhydroxide [AlO(OH)] NPs effectively escaped from the endosome to the cytosol by causing lysosomal swelling and damage. Further, lymph node targeting and long-term retention were achieved using 90 nm-sized nanoparticulate AlO(OH) adjuvants; this led to an effective CD8+ T cell response in vivo (Figure 4A). Another study reported that the shape, crystallinity, and surface hydroxyl group display of AlO(OH) NP adjuvants regulated NLRP3 inflammasome stimulation in human THP-1 myeloid cells or murine bone marrow-derived dendritic cells (Figure 4B) [131,134]. These results show that the nano-engineered design of aluminum-based adjuvants can be used to develop more potent next-generation vaccines.

Modern adjuvants are being incorporated into NP design processes, though their presence is primarily motivated by the need to improve cellular immune responses and suppress lytic properties in physiological environments. Quillaja saponin-based adjuvants, immune stimulating complexes (ISCOMs), and Matrix-M^TM^ consist of 40 nm cage-like nanostructures comprising cholesterol and phospholipids [135,136,137,138]. These adjuvants induce Th1 and Th2 responses to activate cytotoxic T lymphocytes, in addition to stimulating a robust antigen-specific humoral response. An evaluation of several inherent immunomodulatory soluble adjuvants demonstrated significant differences between Matrix-M^TM^ and other evaluated adjuvants. Matrix-M^TM^ induced a significantly higher level of T cell co-stimulatory molecules CD86 and CD69 in activated dendritic cells located in draining lymph nodes. Further, vaccination using an influenza antigen adjuvanted with Matrix-M^TM^ led to the induction of expression of a broad range of cytokines (IL-2, IFN-γ, IL-1β, TNF, IL-4, IL-5, IL-10, keratinocyte-derived cytokine, and IL-12), a re-stimulation response, and balanced antigen-specific IgG1 and IgG2a responses in mice. ISCOM- and Matrix-M^TM^-formulated vaccines have been successfully used in animals for many years and are currently being tested in human clinical trials against COVID-19, seasonal influenza, respiratory syncytial virus, and Ebola virus.

### 3.4. Future Directions of Vaccine Development Using Nanoplatforms

Vaccines play a key role in protecting humans from emerging infectious diseases. Currently, in the case of COVID-19, the continuous appearance of viral mutants since the first outbreak in 2019 and breakthrough infections after vaccinations are major issues. A broad range of novel or next-generation vaccine antigens, such as mRNA, DNA, and synthetic and recombinant components, are being developed and used instead of microbial-derived components. These types of components show clear advantages over conventional classical antigens from a manufacturing and process control standpoint. As novel antigens have successfully completed clinical trials and have been approved for human use, antigens tailored to emerging infectious diseases will continue to be widely proposed. Future vaccine research requires technology that can be applied immediately to clinical and preclinical experiments, with easy production when a viral disease emerges. Nanoengineering is being applied to overcome major issues associated with recombinant protein subunit, mRNA, and DNA vaccines that have potential for clinical application. These issues include poor immunogenicity and insufficient induction of inappropriate immune responses. Nanoparticulate vaccines are primarily being developed to safely deliver antigens to APCs and to maximize immune activity by protecting against proteolysis in the physiological environment. Nanoplatforms can be optimally engineered for various molecules and specific antigens by modulating the physicochemical properties of their components or by presenting multimeric forms conjugated or formulated with immune potentiators. Further, it is important to develop systems that use antigens and molecules encapsulated within NPs to deliver them to the desired location with appropriate timing; it is also important to ensure tightly controlled release kinetics. Consequently, nanoengineered vaccines have great potential to be a flexible platform and provide novel strategies for the development of the next generation of vaccines.

## 4. Treatment

The development of effective antiviral agents is essential for treating or alleviating severe symptoms and preventing death in infected patients. Timely antiviral therapy is an important measure to reduce the burden on the health care system. A variety of synthetic and natural antiviral agents have been developed, including chemical compounds, peptides, and essential oils. These agents exhibit antiviral activity against various types of viruses [139]. Other FDA-approved therapeutics, such as oseltamivir, zanamivir, and abacavir, are being utilized in antiviral therapy for influenza and HIV infection [140]. Remdesivir is now being used for the treatment of COVID-19, and its usage is associated with a significant reduction in the mortality of infected patients [141]. Despite the contributions of antiviral agents, there are several challenges associated with their usage, such as limited efficacy because of poor solubility, low biostability, and toxicity. Additionally, improvements can be made regarding the therapeutic effects of antiviral agents, which mostly block viral proteins and cellular receptors involved in viral infection pathways, such as site-specific delivery of antiviral agents to enhance efficacy and reduce off-target effects. Thus, there is a need for efficient delivery of antiviral agents [142].

### 4.1. Delivery

**Figure 5:**
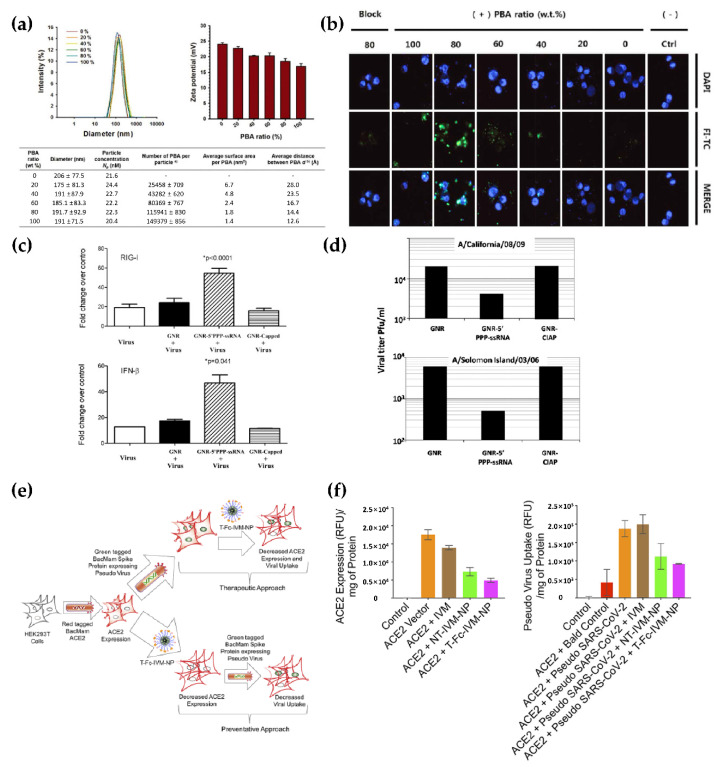
(**a**) Characterization of phenyl boronic acid (PBA)-conjugated polymersome (PBASome)-controlled inter-ligand distance between PBA conjugates. (**b**) Confocal laser scanning microscopy (CLSM) images of PBASomes in the presence of various weight fractions, as observed when stained for lectin. Green, fluorescein isothiocyanate; blue, DAPI; red, WGA. Reproduced with permission from [146]. Copyright (2018) Royal Society of Chemistry. (**c**) Enhanced expression of RIG-I and IFN-β in A459 cells treated with GNR-5′PPP-ssRNA indicates inhibition of infection by H1N1 influenza virus and Solon Islands seasonal flu strain. (**d**) Inhibition of viral replication of the 2009 pandemic H1N1 influenza virus and Solomon Islands seasonal flu strain in the presence of GNR-5′PPP-ssRNA, evaluated in A459 cell lines. Reproduced with permission from [169]. Copyright (2010) National Academy of Sciences. (**e**) Schematic of the mechanism of T-Fc-IVM-NP in HEK293T via decreased ACE2 expression and viral uptake. (**f**) Inhibition of ACE2 expression and pseudovirus uptake in HEK293T cells were evaluated using therapeutic (left) and preventative (right) treatment methods. Reproduced with permission from [170]. Copyright (2020) American Chemical Society.

To address the aforementioned issues, nanoparticulate delivery systems have been applied in the development of antiviral treatments. These delivery systems ensure stable systemic circulation and sustained release of antiviral agents, thus optimizing therapeutic effects [143]. Various types of nanomaterials have been utilized to develop nanoscale delivery systems, and the properties of these nanomaterials can be adjusted by controlling the size of the system, modulating its surface charge, and conjugating targeting moieties to the surface (Table 2). A wide range of nanomaterials, such as LNPs [144], polymeric NPs, AuNPs [145], and silver NPs (AgNPs), are commonly used as carriers for entrapped antiviral agents. These carriers can be easily functionalized with targeting moieties, including antibodies, ligands, and receptors. A delivery system using bilayer polymeric vesicles functionalized with phenylboronic acid (PBA) has been reported for IAV treatment [146]; the surface density of the cell-targeting moiety was modified for improved performance (Figure 5A). The optimal ratio of the carrier and PBA (which interacts with the cell receptor sialic acid) was carefully evaluated via in vitro studies, which led to increased cellular uptake of the antiviral agents mir-323a and favipiravir that were co-loaded in the carrier (Figure 5B). Polymeric vesicles functionalized with PBA enhanced the therapeutic effect of the antiviral agents and preserved cell viability to a greater extent than the free drug (Figure 5C). This demonstrated the synergistic therapeutic effects and significantly enhanced biocompatibility of these systems. The synergistic therapeutic effects associated with dual-delivery were further explored to inhibit the entry of HIV-1 [147]. Liposomes constructed with 1-palmitoyl-2-oleoyl-sn-glycero-3-phosphocholine and 1,2-dipalmitoyl-sn-glycero-3-phosphoethanolamine-*N*-[methoxy-(poly(ethylene glycol))-2000] successfully encapsulated two clinically relevant entry inhibitors: enfuvirtide and protoporphyrin IX. Dual-loaded liposomal delivery carriers enabled enhanced antiviral activity against HIV-1 compared to single-loaded carriers. These results highlight the advantages of employing efficient delivery systems that elicit synergistic effects to inhibit HIV-1. The delivery of dual-loaded inhibitors in a single carrier with high efficiency was the key factor in this regard.

Tuning the surface charge of the delivery system provides an attractive strategy for improving delivery. It assists in increasing the cellular uptake of NPs and facilitates the loading of negatively charged nucleic acid therapeutics [168]. AuNPs are especially noteworthy in this regard and have been used frequently in biomedical applications because of their amenability to surface modification. Gold nanorods (GNRs) modified with cationic charges were reported to function as stable delivery carriers, facilitating electrostatic interactions with anionic antiviral agents against pandemic H1N1 influenza virus [169]. GNR nanoplexes, consisting of GNRs and a genetic agent [5′PPP-ssRNA; ligand of retinoic acid-inducible gene I (RIG-I)] that activates antiviral signaling pathways in respiratory epithelial cells, showed cellular internalization comparable to that of commercially used agents (Figure 5D). The expression of RIG-I and IFN-β is associated with immune responses and the inhibition of viral replication. The expression of these molecules was significantly increased by GNR nanoplexes, ultimately reducing the replication of both seasonal and pandemic H1N1 influenza viruses (Figure 5E).

Nanoparticulate delivery systems can be administered using various delivery routes, which allows for site-specific delivery of these systems. Thus, the delivery routes can be optimized based on the mechanism of action of the antiviral agents. The intravenous route is commonly used because of its effectiveness in emergencies; however, this route has several disadvantages, such as high cost and inconvenience associated with injections. Non-invasive oral administration has been studied as an alternative [171]. Dhar et al. investigated an orally administrable nanoparticulate system encapsulating the clinically approved antiviral drug ivermectin (IVM) for the treatment of COVID-19 [170]. A nanocarrier was constructed with poly (lactide-co-glycolide)-b-poly (ethylene glycol)-maleimide (PLGA-b-PEG-Mal) and the Fc immunoglobulin fragment; it elicited a FcRn-driven crossing of the epithelial barrier in the intestine, to access the bloodstream. The NP-mediated delivery of IVM facilitated sustained release, enabling the maintenance of an optimal concentration of the drug. This significantly improved prophylactic efficacy by suppressing the expression of ACE2, which contributes to viral entry (Figure 5F,G). The aforementioned delivery system demonstrated potential as an effective antiviral platform for the treatment of COVID-19.

### 4.2. Viral Inhibition

Inhibition of viral replication is crucial for antiviral treatment and many antiviral agents have been developed in this regard. Nanoparticulate systems are primarily used to complement antiviral agents as delivery carriers, to improve therapeutic efficacy [172]. Using an understanding of the fundamentals of NPs, nanoparticulate systems capable of inhibiting viral replication have been developed and have been strategically designed to block certain viral replication stages, such as receptor binding, cellular entry, and translation of viral proteins [154].

**Figure 6:**
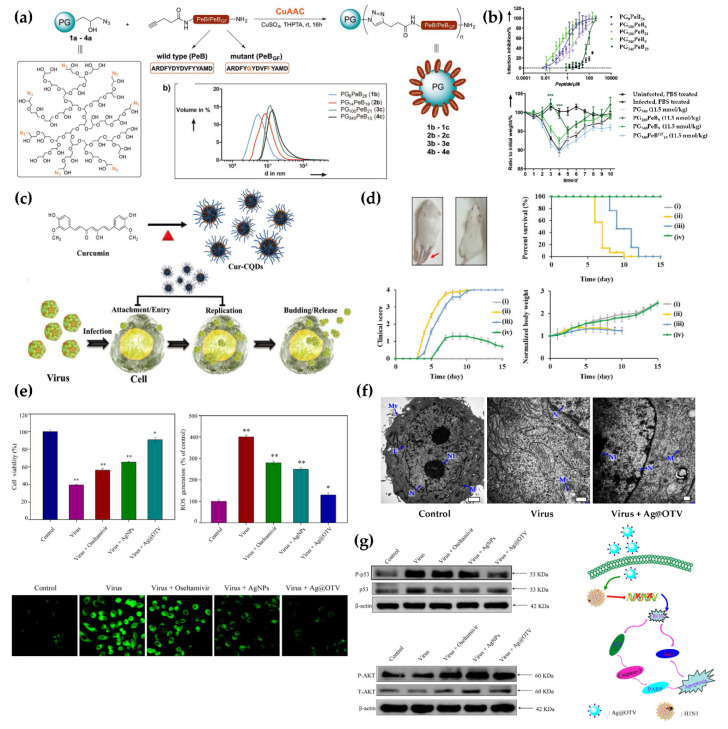
(**a**) Scheme for polymeric nanoparticle (PGs) conjugated with peptide (PeB) in various molecular weights is represented. (**b**) Inhibition of influenza A virus infection by PG-PeBs, prepared using different molecular weights of PeBs, was evaluated in vitro (top) and in vivo (bottom) (*** *p* < 0.001). Reproduced with permission from [175]. Copyright (2017) Wiley. (**c**) Schematics showing the synthetic process of curcumin-derived copper QDs (Cur-CQDs) (top) and their mechanism of antiviral activity (bottom). (**d**) Therapeutic effect of Cur-CQDs against enterovirus 71 (EV71) infection was evaluated in an in vivo study. Survival rates, clinical scores, and body weights of (i) mice without infection and mice injected with (ii) PBS, (iii) curcumin, or (iv) Cur-CQDs, followed by challenge with EV71. Reproduced with permission from [179]. Copyright (2019) Wiley. (**e**) Antiviral activity of silver nanoparticles decorated with antiviral agents (Ag@OTV) was evaluated by determining the viability of H1N1-infected cells (top) and analyzing the ROS production detected based on DCF fluorescence intensity (bottom) in H1N1-infected cells. One-way analysis of variance (ANOVA) was used for statistical analysis. Data represent mean ± standard deviation (* *p* < 0.05 or ** *p* < 0.01) (**f**) TEM image cell sections treated with control media (left), virus (middle), and virus + Ag@OTV (right), where the scale bars were 1 μm (left), 200 nm (middle), and 200 nm respectively. (**g**) ROS-mediated apoptotic signaling pathway, as well as p53 and AKT signaling pathways, are regulated by Ag@OTV. Reproduced with permission from [181]. Copyright (2016) American Chemical Society.

Receptor binding is the first stage of the virus life cycle and is an effective target for nanoparticulate systems. Recently, it has been reported that NPs prepared via facile surface modulation showed strong binding with viruses. This prevented the viruses from accessing the cell receptor, resulting in the effective blockage of viral replication [160,173]. For example, Nie et al. reported that spiky nanostructures (SNSs) with geometry-matching topography inhibit the IAV [174]. SNSs, consisting of silica NPs (SNPs), were designed to match the size and topography of IAV and were coated with a erythrocyte membrane to target the hemagglutinin of IAV. SNSs allow for strong binding to IAV and block viral entry into the cells, thereby reducing up to 85% of cellular infections. This study provided a potential feature, the topography of NPs, to consider during the development of viral inhibitors. The antiviral activity of mesoporous SNPs grafted with distinct organic groups, such as aminopropyl, glycidyloxypropyl, or phenylethyl groups, was investigated against HIV [160]. Various SNPs with distinctive colloidal properties can be produced by balancing the hydrophilicity and hydrophobicity during the surface modification process. SNPs also have different virucidal activity and cytotoxicity profiles, depending on the surface-grafted chemicals. Peptide-polymer NPs with an affinity for influenza virus were evaluated for effective receptor blocking by adjusting the peptide density (Figure 6A) [175]. Biocompatible polyglycerol NPs decorated with a peptide (PeB) at a high density exhibited a strong affinity for the influenza virus via multivalent binding, eliciting significant inhibition of influenza virus infections (Figure 6B). These results highlight that tuning the surface properties of NPs may potentially help in controlling viral transduction; these NPs can be used as antiviral agents and as delivery carriers for antiviral treatments. The synergistic antiviral effects of curcumin-modified AgNPs (cAgNPs) were investigated for the efficient inhibition of respiratory syncytial virus (RSV) infections [176]. The poor water solubility of curcumin and the toxicity of AgNPs were effectively mitigated by combining these two agents. Additionally, as both agents have virucidal properties, the combination system exhibited remarkable antiviral activity against RSV infections without notable cytotoxicity. Regarding viral inhibition, biocompatible cell-mimicking nanodecoys derived from cells have been reported [177]. Rao et al. developed nanodecoys with genetically engineered human embryonic kidney 293T/ACE2 cells for treating COVID-19. The nanodecoys with ACE2 and cytokine receptors significantly inhibited viral replication and infection by competing with host cells for virus and cytokine binding. Nanodecoys enable customization with various types of cellular vesicle components. Li et al. reported that ACE2 nanodecoys derived from human lung spheroid cells (LSCs) neutralized COVID-19 and mitigated lung injury [178]. Furthermore, LSC-nanodecoys promoted viral clearance and reduced lung injury via inhalation administration.

Controlling the redox environment in the host cell reportedly provides control over viral replication in the cell. Reactive oxygen species (ROS) are representative messengers of cell signaling and can trigger inflammatory signaling pathways and apoptosis in virus-infected cells. A ROS-scavenging system using curcumin-derived copper QDs (Cur-CQDs) has been developed to inhibit enterovirus 71 [179]. The CQD component of Cur-CQDs regulated ROS levels, while the curcumin component blocked viral entry, leading to the effective suppression of viral replication (Figure 6C). Polymeric NPs constructed with conductive polymers have also been used to inhibit viral replication by regulating ROS levels [88]. This polymeric nano-regulator successfully decreased ROS levels in cells incubated with various types of influenza viruses, including H1N1, H3N2, and H9N2, without significantly affecting the viability of the infected cells (Figure 6D). Modulation of ROS can also affect the apoptosis pathways of infected cells; these pathways are essential for the proliferation of viruses. Decreasing cell death is a different approach for developing antiviral therapy. Based on this strategy, nanoparticulate systems co-employing antiviral therapeutics with viral binding properties and NPs with antiapoptotic properties have been developed [180]. For example, AgNPs decorated with antiviral therapeutics, such as oseltamivir [181] and zanamivir [182] (targeting the influenza virus), potentiated the inhibition of viral replication in, and apoptosis of, the infected cell. These results revealed improved cellular uptake and similar inhibition of influenza virus infections (Figure 6E–G). Consequently, AgNPs associated with antiviral therapeutics significantly decreased the apoptosis of H1N1 influenza virus-infected cells by regulating ROS levels, demonstrating the potential application of such NPs in antiviral therapy.

Therefore, nanomaterials possess virucidal potential and provide novel routes for the development of antiviral systems utilizing the dual functionality of NPs. Antiviral nano-systems can be applied as antiviral barriers to coat frequently contacted surfaces, such as masks, public areas, and healthcare infrastructure [183]. The use of antiviral nanomaterials, including copper [184], graphene oxide [168], zinc oxide [185], and AgNPs [186], has shown potential in preventing the spread of viruses through contact transmission. Antiviral nanomaterials may play a pivotal role in the development of practical measures to prevent future outbreaks.

## 5. Conclusions

Viruses have appeared in the past and will continue to appear in the future. As is currently being observed, a single pandemic can threaten the lives of many and create numerous economic and social challenges. The need for an effective solution to this problem has now been fully recognized. The nano-sized nature of viruses suggests that nano-sized technological solutions may be needed to successfully combat these agents. Consequently, nanotechnology research for the development of prophylactic vaccines, diagnostics, and therapeutics against infectious pathogens is likely to continue advancing in the future.

Studies on diagnostic technologies are essential for combating emerging and re-emerging viruses, as well as for the rapid control and prevention of illness and death. Nanomaterials may improve the sensitivity of existing diagnostic technologies because of their controllable properties, such as high reactivity, adsorption capacity, particle size, and physicochemical bonding capacity. In addition, application of NPs may lead to improvements in technology, with respect to cost, time, specificity, mobility, and convenience. Studies using nanomaterials to identify transmission patterns of infectious diseases and the mechanisms underlying virus infections, to apply NPs to virus diagnostic technologies, are being actively proposed and carried out. Thus, using nanotechnology-based innovations, it is possible to establish a predictive and early monitoring system that will help prevent the spread of viral diseases; additionally, these innovations will aid in the rapid and accurate diagnosis of infected individuals.

Safety concerns will be the most important issues concerning the development of nanotechnology-based vaccines and anti-infective therapy. Potentially severe adverse effects may include anaphylaxis, pyrogenic fever responses, organ-specific toxicity, and immune-mediated toxicities (e.g., immune activation or suppression and autoimmune diseases induced by excessive cytokine release). Therefore, adequate information regarding the pharmacological and toxicological effects of the prophylactic and therapeutic agents should be available prior to the initiation of clinical research and product development. Under the EUA approval, some nanomaterials are being applied to humans to combat the COVID-19 pandemic. It is certain that the number and speed of approvals for the use of nanomaterials in humans will increase in the future. The implementation of nanomaterial-based prophylactic and therapeutic systems with appropriate safety profiles will contribute to being suitably prepared for combating and managing emerging infectious diseases.

Although further research is needed regarding the clinical use of nanomaterials, the prophylactic and therapeutic effects of existing modalities have been clearly improved by the application of NP-based delivery systems. Further, the incorporation of nano-engineered materials has led to an increase in the sensitivity and speed, as well as a reduction in the cost, of existing diagnostic methods. Innovations in the usage of nanomaterials in diagnostic systems, vaccines, and treatment modalities will continue to advance and will ultimately help combat infectious disease pandemics.

## Figures and Tables

**Table 1:** Nanomaterial-based diagnostics for emerging and re-emerging viral diseases.

Nanomaterials	Diagnostic Techniques	Target	LOD	Time	Ref.
Carbon nanotubes	RDT	DENV	8.4 × 102 TCID50/mL	>10 min	[6]
		SARS-CoV-2	0.55 fg/mL	>5 min	[7]
	Immunological	Influenza A Virus (H1N1)	1 PFU/ml	30 min	[8]
Graphene	Immunological	JEV/AIV	1 fM/10 fM	1 h	[9]
		AIV	1.6 pg/mL	30 min	[10]
		HIV-1	2.3 × 10^−14^ M	1 h	[11]
		Influenza A Virus (H5N1)	25 PFU/mL	15 min	[12]
		Zika Virus	450 pmol/L	5 min	[13]
AuNPs	Optical	SARS-CoV-2	0.18 ng/μL	>10 min	[14]
			50 RNA copies per reaction	30 min	[15]
			4 copies/μL	40 min	[16]
		Hepatitis B virus	100 fg/mL	10–15 min	[17]
	Immunological	SARS-CoV-2	370 vp/mL	15 min	[18]
			0.08 ng/mL	30 min	[19]
		Influenza A Virus	7.8 HAU	30 min	[20]
		Zika Virus	0.82 pmol/L	50 min	[21]
Quantum dots	ELISA	Influenza A Virus	22 pfu/mL	>35 min	[22]
		Influenza A Virus (H5N1)	0.016 HAU	>15 min	[23]
		SARS-CoV-2	5 pg/mL	>15 min	[24]
	Immunological	HEV3	1.23 fM	20 min	[25]
		SARS-CoV	0.1 pg/mL	1 h	[26]
Synthetic polymer	Immunological	Influenza A Virus (H1N1)	5 × 10^3^~10^4^ TCID50	9 min	[27]
Nanofibers	Immunological	SARS-CoV-2	0.8 pg/mL	20 min	[28]

Abbreviations: RDT: rapid diagnosis test, DENV: dengue virus, JEV: Japanese encephalitis virus, AIV: avian influenza virus, HEV3: hepatitis E virus 3, AuNP: gold nanoparticle, HIV: human immunodeficiency virus, ELISA: enzyme-linked immunosorbent assay, LOD: limit of detection, SARS-CoV-2: severe acute respiratory syndrome coronavirus 2, TCID50: 50% tissue culture infectious dose, HAU: haemagglutinin unit.

**Table 2:** Nanomaterials for antiviral therapy.

Nanoparticles	Drugs	Target Virus	Strategy	Level of Study	Advantages in Antiviral Therapy	Ref.
Cellulose PEG NPs	Zidovudine	HIV	Inhibition of viral replication	In vitro	Improved encapsulation efficiency Targeted delivery with sustained release	[148]
Lipid PLGA NPs	Latency -reversing agents	HIV	Inhibition of viral replication	In vitro	Synergistic latency-reversal and low toxicity	[149]
PVP/SA PEG NPs	Zidovudine	HIV	Inhibition of viral replication	In vitro	Improved cellular internalization	[150]
PLA NPs	Chloroquine	HSV-1	Blocking viral entry	In vitro	Targeted delivery with sustained release	[151]
Lipid nanodisc		IAV	Viral inactivation	In vitro In vivo	60% reduction of viral infection 40% reduction of death rate in mice	[152]
AuNPs		IAV	Inhibition of viral infection	In vitro	40% reduction of viral infection	[153]
		RSV, VSV HPV, dengue	Viral inactivation	In vivo Ex vivo	87% inactivation of virus	[154]
GO-AgNPs		FCoV IBDV	Blocking viral entry	In vitro	25% inhibition of FCoV infection 23% inhibition of IBDV infection	[155]
AgNPs		TGEV-CoV	Inhibition of viral replication	In vitro	67.35% reduction of viral replication	[156]
		RSV	Inhibition of viral replication	In vitro In vivo	75% reduction of viral replication	[157]
SeNPs	Zanamivir	IAV	Inhibition of viral infection	In vitro	Improved infected cell viability up to 73%	[158]
ZnO NPs	Oseltamivir	IAV	Viral inactivation	In vitro	Improved infected cell viability up to 90%	[159]
SiO_2_NPs		HIV VSV	Inhibition of viral infection	In vitro	50% reduction of viral infection	[160]
FE_3_O_4_@SiO_2_		HSV	Viral inactivation	In vitro	Improved antiviral activity	[161]
Ag_2_S	Glutathione	PEDV-CoV model	Inhibition of viral replication	In vitro	Reduction of viral titer	[162]
CDs		HCoV-229E	Blocking viral entry	In vitro	CD-mediated inhibition of viral entry	[163]
		PEDV-CoV Model	Inhibition of viral replication	In vitro	80% reduction of viral replication	[164]
		PRRSV-CoV Model	Viral Inactivation	In vitro	Reduction of viral replication	[165]
		Zika, dengue	Inhibition of viral infection	In vitro	Improved infected cell viability up to 90%	[166]
Cellulose nanocrystals		Alphavirus	Inhibition of viral infection	In vitro	100% inhibition of viral infection	[167,168]

Abbreviations: PEG: poly (ethylene glycol), PLGA: polylactide-co-glycolide, PVP: Poly (vinyl pyrrolidone)-, SA: sialic acid, PLA: poly (lactic) acid, HSV: herpes simplex virus, IAV: influenza A virus, CD: carbon dot, VSV: vesicular stomatitis virus, HPV: human papillomavirus, RSV: respiratory syncytial virus, GO: graphene oxide, IBDV: infectious bursal diseases virus, FCoV: feline coronavirus, AuNP: gold nanoparticle, AgNP: silver nanoparticle, HIV: human immunodeficiency virus, PEDV: porcine epidemic diarrhea virus, PRRSV: porcine reproductive and respiratory syndrome virus, TGEV: transmissible gastroenteritis coronavirus, NP: nanoparticle.

## Data Availability

This study did not report any data.
